# Clinicopathological study of gastric schwannoma and review of related literature

**DOI:** 10.1186/s12893-022-01613-z

**Published:** 2022-05-10

**Authors:** Zhihan Zhong, Yuhao Xu, Junwei Liu, Chengwu Zhang, Zunqiang Xiao, Yan Xia, Yu Wang, Jianfeng Wang, Qiuran Xu, Yi Lu

**Affiliations:** 1 The Second School of Clinical Medicine, Zhejiang Chinese Medical University, Hangzhou, 310053 China; 2 General Surgery, Cancer Center, Department of Hepatobiliary & Pancreatic Surgery and Minimally Invasive Surgery, Zhejiang Provincial People’s Hospital (Affiliated People’s Hospital, Hangzhou Medical College), Hangzhou, Zhejiang China 310014; 3 Cancer Center, Department of Pathology, Zhejiang Provincial People’s Hospital (Affiliated People’s Hospital, Hangzhou Medical College), Hangzhou, 310014 Zhejiang China; 4 Medical Record Department, Zhejiang Provincial People’s Hospital, Affiliated People’s Hospital, Hangzhou Medical College, Hangzhou, Zhejiang China; 5 Department of Cardiology, Chunan Chinese Traditional Medicine, Hangzhou, Zhejiang China; 6 Laboratory of Tumor Molecular Diagnosis and Individualized Medicine of Zhejiang Province, Zhejiang Provincial People’s Hospital, Affiliated People’s Hospital, Hangzhou Medical College, Hangzhou, China

**Keywords:** Schwannoma, Stomach, S100 protein, Prognosis

## Abstract

**Background:**

This study aimed to investigate the clinical features, diagnostic criteria, treatment options, and prognosis of patients with gastric schwannoma (GS).

**Methods:**

We collected the clinical data of all patients pathologically diagnosed with GS in Zhejiang Provincial People's Hospital from May 2012 to October 2021.

**Results:**

A total of 26 cases of GS were analyzed clinicopathologically, where the sizes of the tumor were found to be in the range of 1–6 cm (mean: 3.16 cm, median: 3.05 cm). A computed tomography (CT) scan analysis revealed that most masses were either moderately progressive or uniformly enhanced. According to ultrasound gastroscopy results, most of them were hypoechoic masses. There were 23 cases of surgery and three cases of endoscopic submucosal tumor dissection. Immunohistochemistry demonstrated that S100 was positive in 26 patients, immunomarker SOX10 was positive in five, whereas CD34, CD117, and SMA were negative in most patients. CK (Pan), Dog-1, and Desmin were also found negative. All 26 cases were followed up after the conclusion of the study where no evidence of recurrence or metastasis was observed.

**Conclusions:**

GS is a unique form of peripheral schwannoma. The diagnosis of this type of tumor depends on the pathology and immunohistochemistry of the individual. The key to treating this type of tumor is endoscopy and surgery. Follow up and related literature review showed that GS was a benign tumor with little possibility of malignant transformation.

## Background

Gastric mesenchymal tumors mainly consist of three types of tumors: gastric stromal tumors, leiomyomas, and schwannomas. Gastrointestinal schwannoma (GS) is a rare tumor that grows slowly in the digestive tract, which is often difficult to be initially diagnosed without surgery; thus, a differentiated pathology and immunohistochemistry are conducted. The most common site of this tumor is known to be the stomach [1]. GS accounts for only 0.2% of all types of gastric tumors, which usually remain asymptomatic and nonmalignant [2]. Even though its endoscopic manifestations and CT findings are similar to that of gastric stromal tumors [3], these preoperative examinations can still be helpful to determine the location, size, and origin of the tumor, as well as whether it is ulcerative or hemorrhagic, which becomes essential to decide the course of its treatment.

Nonetheless, as the mass enlarges, there is an increased risk of ulcer bleeding, in addition, to an increase in the compression blocking of the gastrointestinal tract. Therefore, active surgical resection is the preferred method of treatment. In this study, we analyzed 26 patients with GS in terms of clinical and imaging features, immunohistochemistry and pathology findings, and relevant literature and conducted further follow-ups to confirm the benign nature of this tumor.

## Materials and methods

This study included 26 patients with pathologically confirmed GS from Zhejiang Provincial People's Hospital between May 2012 and October 2021. The investigators obtained basic information through medical records such as demographics and clinical data, including imaging contrast, treatment methods, and pathological and immunohistochemical data. The rest of the examinations were followed up by telephone, which was essential to conclude the results of this study. Hematoxylin and eosin stains for all patients were reviewed. The immunohistochemical analysis using the anti-biotin protein biotin complex immunoperoxidase technique was conducted.

## Results

The patients included in the study were of age in the range of 35 to 80 years (mean: 59 years; standard deviation: 10.82), consisting of nine men and 17 women, with a male to female ratio of approximately 1:2. The clinical data of recruited patients are summarized in Table 1.

**Table 1:** Clinicopathologic summary of 26 gastric schwannomas

Variables	Case N(%)		Mean	Standard deviation
Age at diagnosis, y			58.92	10.82
< 60	12 (46.15%)		49.25	6.20
> 60	14 (53.85%)		67.21	5.67
Gender				
Male	9 (34.62%)			
Female	17 (65.38%)			
Clinical presentation				
Simple epigastric pain or discomfort	15 (57.69%)			
Abdominal discomfort with nausea	1 (3.85%)			
Abdominal discomfort with black stool	1 (3.85%)			
Simple black stool	1 (3.85%)			
Acid reflux	1 (3.85%)			
No symptoms	7 (26.92%)			
Outcome				
Rechecked regularly and had no recurrence or metastasis	19 (73.08%)			
Not rechecked and never complained of any obvious discomfort	7 (26.92%)			
Tumor size(cm)			3.14	1.40
≤ 2 cm	7 (26.92%)		1.49	1.50
> 2 cm, ≤ 3 cm	6 (23.08%)		2.65	2.75
> 3 cm, ≤ 4 cm	8 (30.77%)		3.61	0.35
> 4 cm, ≤ 5 cm	2 (7.69%)		4.70	0.14
> 5 cm	3 (11.54%)		5.67	0.42
Ulcer				
Yes	7 (26.92%)			
No	19 (73.08%)			
Bleeding				
Yes	2 (7.69%)			
No	24 (92.31%)			
CT enhancement pattern of mass				
Substantial enhancement	4 (18.18%)			
Moderate progressive enhancement	3 (13.64%)			
Moderate uniform enhancement	6 (27.27%)			
Moderate uneven enhancement	2 (9.09%)			
Mild enhancement	1 (4.55%)			
Undescribed enhancement mode	6 (27.27%)			
Lack	4 (18.18%)			
Mass site				
Gastric body	19 (73.08%)			
Gastric antrum	4 (15.38%)			
Gastric angle	2 (7.69%)			
Gastric cardia	1 (3.85%)			
Mass origin				
Muscularis propria	24 (92.31%)			
Submucosa	2 (7.69%)			
Condition of echo				
Low echo	21 (80.77%)			
Meduim echo	1 (3.85%)			
High echo	1 (3.85%)			
No echo description	3 (11.54%)			

Among the 26 cases, one or more presenting complaints were documented. Simple epigastric pain or discomfort (n = 15), abdominal discomfort with nausea (n = 1), abdominal discomfort with black stool (n = 1), simple black stool (n = 1), and acid reflux (n = 1) were the most frequently reported complaints. Seven patients were admitted to the hospital despite having no symptoms and treated for unrelated medical procedures detected during a CT scan or gastroscopy.

Several tumor markers were within the normal range in 21 patients. Two cases had cytokeratin 19 levels slightly higher, whereas two cases had slightly higher total PSA levels. All other tumor markers were within the usual range as the patients had no history of the tumor. Neither neurofibromatosis syndrome type 1 nor type 2 has ever been diagnosed in any of the patients.

Interventions in the form of surgery including laparoscopic gastrectomy (n = 22), endoscopic submucosal dissection (n = 3), and robotic gastrectomy (n = 1) were performed in this study.

### Follow-up

After the study was concluded, all 26 patients were followed up for 1 to 9 years (mean 4 years, median 3 years) without significant metastasis or recurrence. Among them, 19 patients were rechecked regularly and had no recurrence or metastasis, while seven patients were not rechecked, and they never complained of any obvious discomfort.

### Gross pathology

**Fig. 1:**
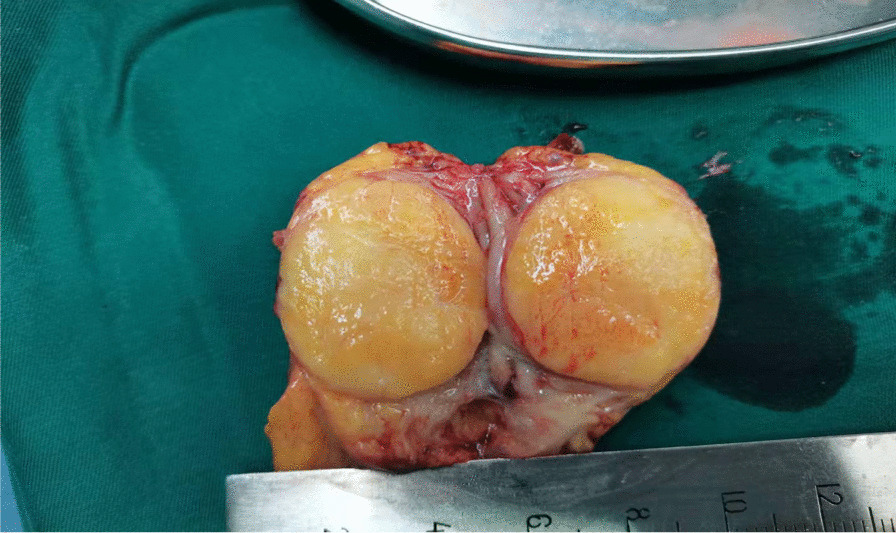
The gross appearance of a case of gastric schwannoma. The surface of the specimen section is grayish yellow

In all cases, the mass was discovered in the following sites: 19 in the gastric body, four in the gastric antrum, two in the gastric angle, and one in the gastric cardia. The mass color was greyish-white and greyish-yellow in 10 cases, greyish-white in seven cases, greyish-yellow in eight cases, and greyish-red in one case. The growth pattern was as follows: 11 cases had protrusions into and out of the cavity, nine cases had protrusions into the cavity, and six cases had protrusions out of the cavity. All tumors had distinct borders; the majority had a medium texture, with three cases having a hard texture. The diameter ranged from 1 to 6 cm (mean: 3.14 cm; standard deviation: 1.40). Ulcer bleeding status observation revealed that 19 cases had no ulcer bleeding, four had only an ulcer, and three had ulcers with bleeding (Fig. 1).

### Imaging results

**Fig. 2:**
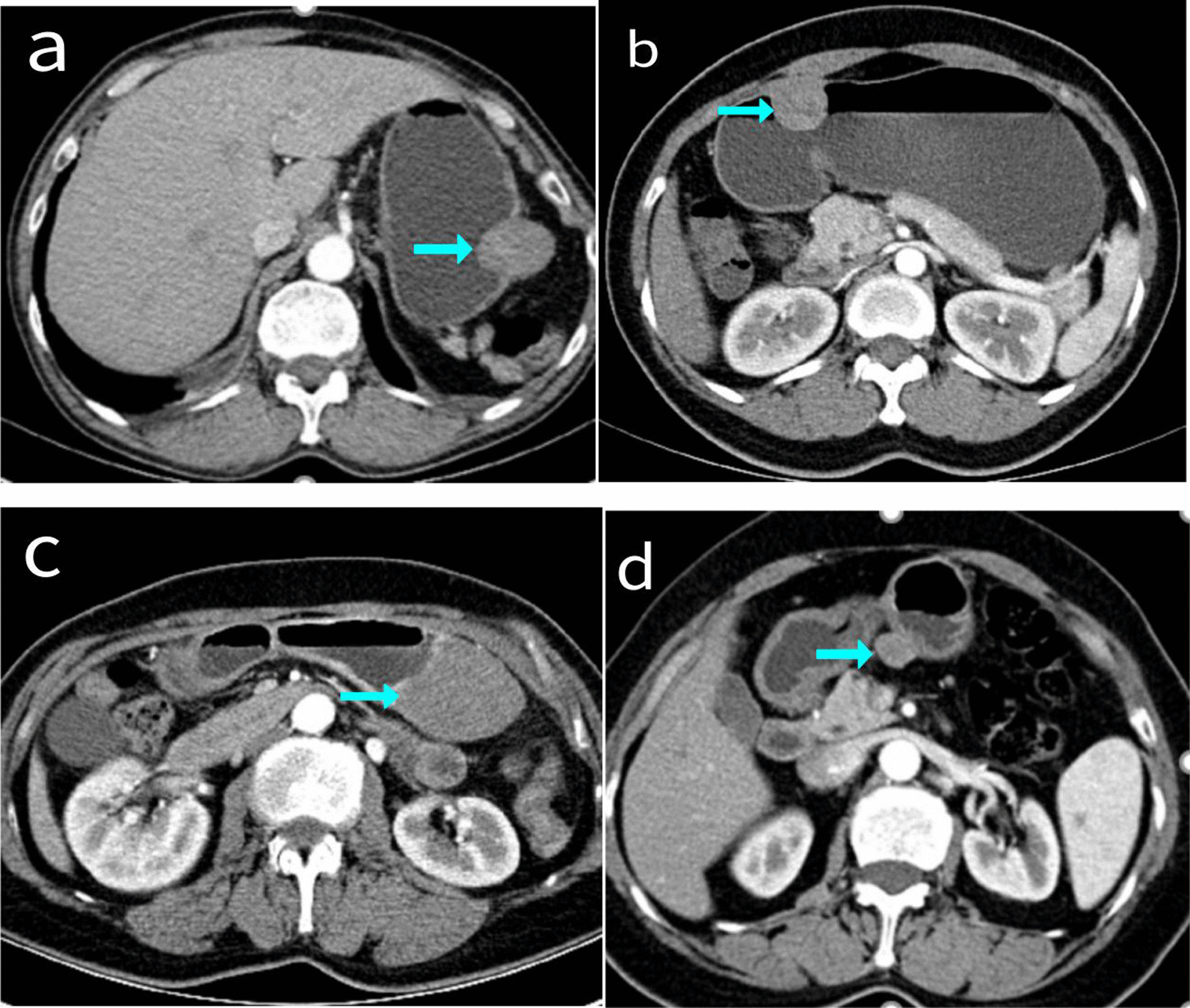
Enhanced CT images of four cases of gastric schwannoma

Among the 26 patients, 22 cases had enhanced CT data (Fig. 2) which revealed that four cases were having substantial enhancement, three had moderate progressive enhancement, six had moderate uniform enhancement, two had moderate uneven enhancement, one had mild enhancement, and six had undescribed enhancement mode. There were no obvious enlarged lymph nodes in 19 of the 22 cases. Only three had multiple enlarged lymph nodes in the hepato-gastric space, gastrocolic ligament area, gastric curvature, and pyloric part. The lymph nodes had a maximum diameter of about 1.7 cm.

**Fig. 3:**
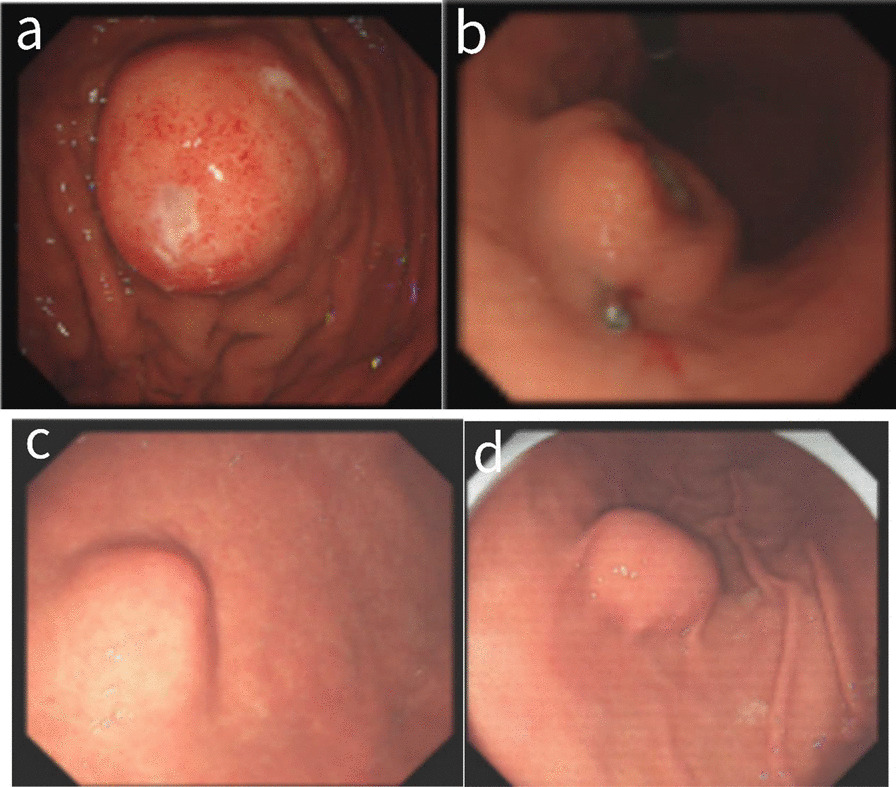
Ultrasonographic gastroscopic images of four cases of gastric schwannoma. Obvious ulcer can be seen on the surface of the mass in the upper two pictures

Ultrasonic gastroscopy was performed on all 26 cases (Fig. 3). Because of the hierarchical etiology of gastric schwannoma (GS), there were 24 cases of muscularis propria and two cases of the submucosa. The echo analysis revealed that 21 cases were low echo, one case was a medium echo, one case was a high echo, and three cases had no echo description (Fig. 3).

### Immunohistochemical findings

**Fig. 4:**
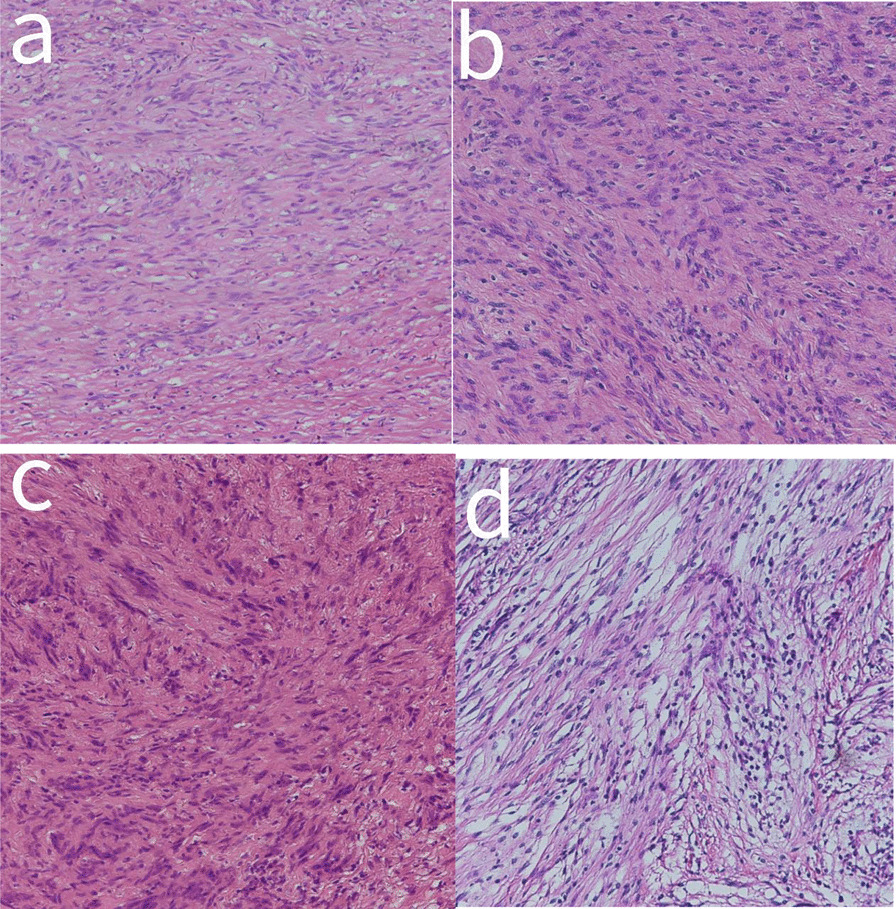
HE piceure of four cases of gastric schwannoma,all of which were spindle cells(All pictures have 10 × 10 magnification)

**Fig. 5:**
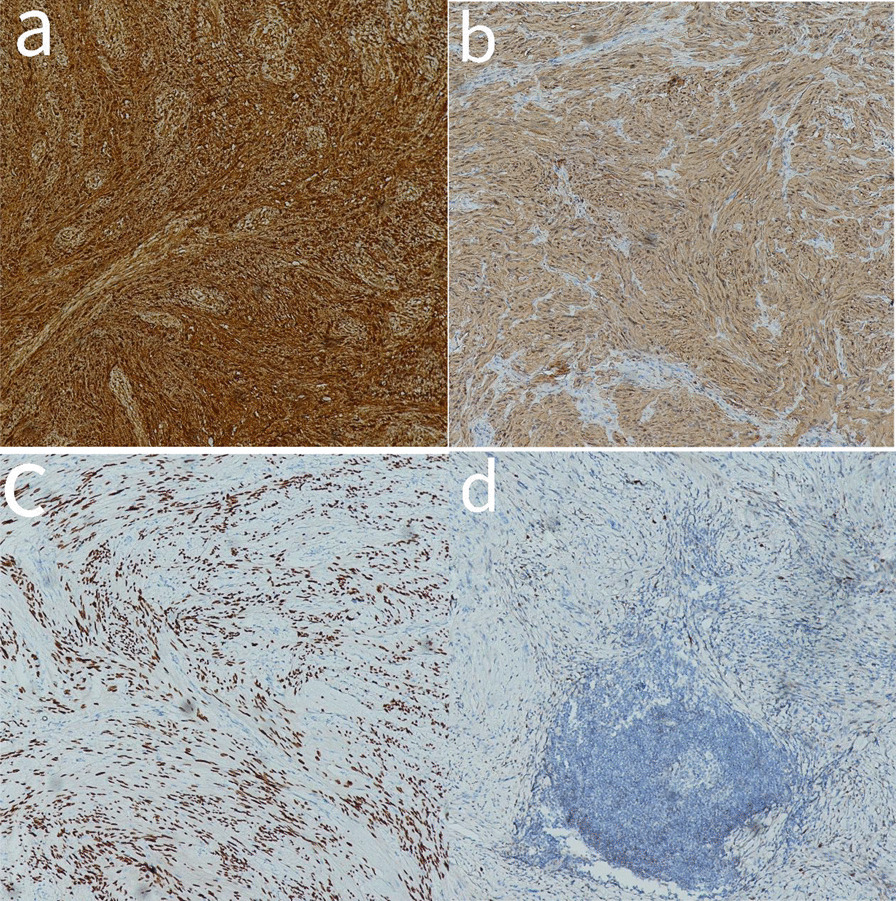
Figure a shows S-100 strong positive; Figure b shows S-100 positive; Figure c shows Sox positive; Figure d shows CD117 negative (All pictures have 10 × 10 magnification)

**Fig. 6:**
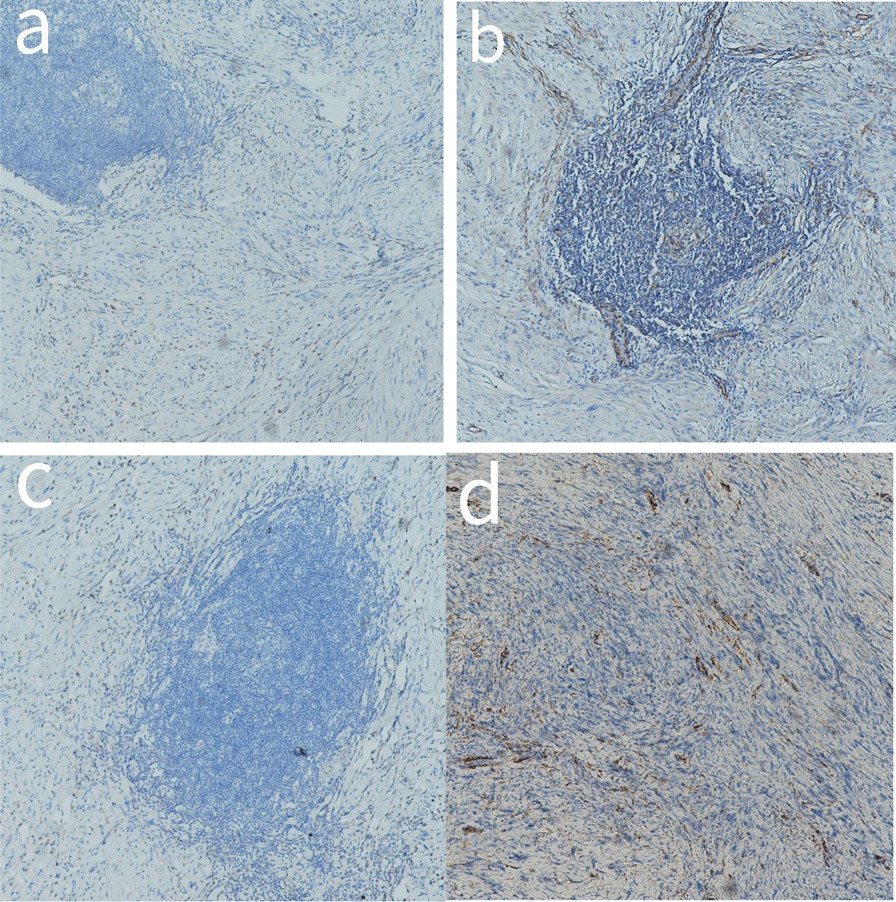
A shows Desmin1 negative; Figure b shows SMA negative; Figure c shows DOG1 negative; Figure d shows CD34 negative (All pictures have 10 × 10 magnification)

The tumors in the case of all 26 patients were spindle cells (Fig. 4). All 26 cases showed positive results for S100 protein, out of which five were strongly positive, whereas SOX 10 was also detected positive in five patients (Fig. 5). Among the 26 cases studied, 23 had varying levels of Ki67, positivity ranging from 1 to 10%, with one case having a 50% positive rate. Most cases (19 out of 26) were negative for CD34, while three were vascular positive and four were partially positive. It also came to our findings that CD117 was negative in most cases (24 out of 26), whereas SMA was negative in a few cases (22 out of 26), and Dog-1 and Desmin were negative in all cases (Fig. 6).

## Discussion

Schwannoma is a stromal tumor that originates from Schwann cells [4]. The occurrence of GS is known to be much less common than gastrointestinal schwannoma (GIST). According to the literature, the prevalence of GS is one in every 45 patients with GIST [5]. It has also been reported that GS is more common in female patients aged 40–60, with a male to female ratio of about 1:2 or higher [6]. The average age of patients in this study was 59 years old, and the male to female ratio was approximately 1:2, which was consistent with the findings of the previous study. The 60% to 70% of GIST is located in the stomach, making it the most common tumor site [7]. Studies have reported that more than half of GS originates from the body of the stomach, while fewer cases come from the antrum or cardia sites [8]. Of 26 studied patients, in 19 (73%), GS was found to originate from the gastric body, with six originating in the gastric horn or antrum and only one originating in the cardia. As the tumor grows slowly, most GS cases (approximately 40%) are asymptomatic and are diagnosed found during a physical examination or unrelated medical procedures [9]. Studies also reported that some patients might be symptomatic and may show indications like abdominal pain, gastrointestinal bleeding, or palpable masses, and a few patients may show gastrointestinal obstruction [6]. In this research, only seven patients were asymptomatic; more than half of the patients experienced abdominal pain or stomach discomfort, whereas two patients experienced gastrointestinal bleeding, manifested as a black stool. Bleeding may occur due to increased submucosal masses, which may affect the blood supply of the overlying mucosa, or because of a decreased gastric acid tolerance [8]. Therefore, the active mode of treatment should be deemed to avoid physical discomfort caused by the mass, ulcer, bleeding, or compression obstruction in GS patients, to improve the quality of life of the patients affected.

In conclusion, because patients with GS do not present with special clinical manifestations or signs, the only way to diagnose GS is through a pathological examination. However, imaging examinations such as enhanced CT and endoscopic ultrasonography (EUS) can also provide helpful information [8]. These examinations can assist in early diagnosis and determining the best course of treatment. EUS is a beneficial technique that can accurately evaluate this type of tumor; it can also roughly determine the nature of the tumor, locate the tumor, define the origin level of the tumor, and whether it is superficial growth or protruding one [10]. In comparison to GIST, GS shows distinct signs, and it appears to be a low-density mass with a clear boundary on EUS compared with the surrounding muscularis propria. It appears to be an uneven hypoechoic lesion, with most of it originating from the muscularis propria [11], whereas more than half of GISTs show enhanced or identical echo [8]. In our study, all masses had distinct borders. Of the 26 patients’ cases, 24 tumors originated from the muscularis propria, and two were found in the submucosa, 21 of which were hypoechoic. However, EUS alone is insufficient for diagnosing GS. All EUS reports in this paper suggest that gastrointestinal stromal tumors should be considered. Although endoscopic ultrasound-guided fine-needle aspiration (EUS-FNA) can assist in a better diagnosis, the National Comprehensive Cancer Network guidelines do not recommend routine EUS-FNA for primary resectable GIST in this condition because of the risk of tumor rupture and spread associated with a poor prognosis [12]. Therefore, routine EUS-FNA for gastric submucosal tumors was not performed in this study.

In CT examination, GS usually shows a clear oval tumor under the gastric mucosa, with an exogenous or mixed growth mode, moderate uniform, or moderate progressive enhancement. Most surrounding lymph nodes do not appear to be enlarged [13], there might be a few of the enlarged perigastric lymph nodes seen that are reactive inflammatory lymph nodes [14]. Hong et al. reported their findings after examining 16 cases of GS at their institution on CT scans, of which 13 cases (81%) showed uniform enhancement [15], and they were all prominent growths, consistent with our results. It was revealed that only three cases had enlargement of the perigastric lymph nodes, and the mode of enhancement was roughly consistent with the literature reviewed above. Still, there were also a few cases of obvious enhancement or uneven enhancement found. In the same way that EUS report was misdiagnosed as GIST, our case too was misdiagnosed as GIST during CT examination. Therefore, additional research is required to improve the positive rate of GS preoperative diagnosis.

Since the average time for GS to amplify is nearly five years [16], surgical resection is the first choice for GS patients due to the risk of obstruction, bleeding, and perforation. Although preoperative imaging cannot provide a definitive diagnosis, it can help determine the mass's location, origin, and size, which helps determine the surgical method and scope of mass resection. Extraluminal growth, central ulcer, and complex site of the lesion (gastric incision or small curvature of upper gastric body) are negative features of endoscopic treatment when the tumor is large (> 5 cm) [17]. When the previously mentioned mass properties were discovered, most experts believed that surgery should be performed. Most other patients in this study had surgical treatment for extraluminal protuberance or mass ulcer, with laparoscopy being the most common treatment method. As technology advances, robot-assisted laparoscopic therapy and endoscopic therapy have emerged as viable treatment options. The advantages of robot-assisted laparoscopic therapy include more precise operation and clearer vision. Therefore, robot-assisted laparoscopic therapy is a safe and appropriate treatment method [18]. Endoscopic resection can also be considered before performing surgery for benign gastric wall masses that may be identified as schwannomas. In three of our cases, endoscopic resection was performed; they had intraluminal protuberant masses in the stomach that were less than 2 cm in diameter, and that originated from the muscularis propria with clear boundaries. There was no ulcer bleeding and no perigastric lymphadenopathy in any of the three patients who underwent the endoscopic resection. Subsequent follow-ups confirmed that none of them had a recurrence or metastasis of their tumor. Based on the experience, we believe that endoscopic resection can be considered when the mass is less than 2 cm in size, originates from the muscularis propria or submucosa, is located on the anterior wall or great curvature of the gastric body does not have ulcer bleeding, and only protrudes into the cavity. Therefore, when performed under the idea of comprehensive preoperative evaluation and controlled surgical pointer, endoscopic mass resection also can cure the disease. In addition, advantages include shortened hospital stay, reduced risk of anesthesia, less severity of pain, less likelihood of postoperative complications, and improved postoperative quality of life after surgery. As the preoperative examination and intraoperative rapid freezing pathology showed benign diseases, all patients followed the CSCO guidelines for diagnosis and treatment of gastrointestinal stromal tumors and underwent R0 tumor resection, whether surgical or endoscopic treatment. Lymph node dissection was performed only in patients with potentially pathologically enlarged lymph nodes.

All cases made a full recovery after the operation. The median follow-up time was three years, and there was no evidence of recurrence. There were 137 cases identified in the relevant literature that were considered, of which 105 cases did not relapse until 36 months after diagnosis, and the remaining cases were related to incomplete resection during tumor treatment [19]. However, 221 cases of schwannoma were reviewed in another paper published in 2017 by Bao Guang Huang et al., including 211 cases of benign schwannoma and 10 cases of malignant schwannoma being identified. The median disease-free survival time for malignant schwannoma was significantly shorter than for benign schwannoma. As a result, we believe that most GS are benign tumors that will not recur, but there are a few malignant possibilities. The histological criteria for the diagnosis of malignant schwannoma are based on mitotic map and the presence of nuclear atypia [20]. When the mitotic rate is greater than 10 / 50 high power field, it needs to be followed up regularly like other malignant tumors [21]. Further research is required to better understand the characteristics of malignant GS [22].

The postoperative pathological and immunohistochemical examination is critical for making the final diagnosis of GS and distinguishing it from stromal tumors and smooth tumors. Typical features of GS immunohistochemistry include positive S100 protein and SOX 10, negative CD34 or only locally positive, and negative Dog-1 [23]. stromal tumors and leiomyomas of the gastrointestinal tract were expressed to have negative S-100, with stromal tumors expressing positive CD34, CD117, and Dog-1, with Dog-1 being the most sensitive [4] and SMA and Desmin being positive in leiomyoma (Fig. 3). S-100 was positive in all cases in this study, while CD34, CD117, and SMA were negative in most cases, and Desmin and Dog-1 were negative.

There are some limitations to our research. First and foremost, our analysis is retrospective and contains some deviations. Second, because such diseases are sporadic, fewer patients are included in the study; however, the number of patients had been in the upper middle range compared to previous studies. Finally, we did not compare GS with other gastric tumor diseases from imaging data.

## Conclusions

In conclusion, we reported 26 patients with gastric schwannoma (GS). This tumor is far less common than GIST, and it primarily affects middle-aged and older women. GS's diagnosis should be made to distinguish it from stromal tumors and smooth tumors. The growth characteristics of the mass and the results of preoperative imaging examination determine the surgical direction and method of resection. R0 resection, i.e., a microscopically margin-negative resection, is the treatment of GS. The diagnosis of this type of tumor depends on the pathology and immunohistochemistry of the individual. Although there is a possibility of tumor progression towards malignancy, much previous literature shows that GS is biologically non-cancerous.

## Data Availability

The datasets used and/or analyzed during the current study available from the corresponding author on reasonable request.

## Abbreviations

CT: Computed tomography
EUS: Endoscopic ultrasonography
EUS-FNA: Endoscopic ultrasound-guided fine-needle aspiration
GS: Gastric schwannoma
GIST: Gastrointestinal schwannoma
